# Vandetanib induced phototoxic reaction progressed to toxic epidermal necrolysis^☆^

**DOI:** 10.1016/j.abd.2021.05.010

**Published:** 2021-11-19

**Authors:** Busra Demirbag Gul, Nilgun Senturk, Deniz Baycelebi, Levent Yildiz

**Affiliations:** a Department of Dermatology, Ondokuz Mayıs University, Faculty of Medicine, Samsun, Turkey; b Department of Pathology, Ondokuz Mayıs University, Faculty of Medicine, Samsun, Turkey

Dear Editor,

Vandetanib is a tyrosine kinase inhibitor that is approved for the treatment of medullary thyroid carcinoma (MTC). As with other tyrosine kinase inhibitors, vandetanib causes various cutaneous side effects like acneiform eruption, rash, and photosensitivity.^1^ Here we describe a patient who developed a phototoxic reaction to vandetanib and then progressed to toxic epidermal necrolysis (TEN) and has been controlled with intravenous immunoglobulin (IVIg). A 43-year-old male was admitted with an erythematous eruption that developed over 3 days. On physical examination, there was a well-demarcated erythematous vesiculobullous rash on the sun-exposed areas of the skin including the face, neck, and hands. The patient had MTC, he was on oral vandetanib therapy (300 mg/d) for 15 days. He denied taking any medication except vandatanib. Few hours prior to the development of the rash, he had been exposed to sunlight for a long time without sun protection. Phototesting and biopsy were not performed. Since the distribution of the eruption was strictly restricted to sun-exposed areas and vandetanib was the only medication, this condition was assumed as a “vandetanib-induced phototoxic reaction”. Vandetanib treatment was stopped. Oral prednisolone 1 mg/kg/day was administered. In a few days, lesions progressed to proximal extremities, back, and chest with the involvement of 30% of body surface area (Figure 1, Figure 2). Oral mucosal erosions and conjunctivitis were observed. Laboratory examination revealed elevated erythrocyte sedimentation rate (65 mm/h) and white blood cells (13.800 mm^3^). Nikolsky's sign was positive. A biopsy was performed and epidermal necrosis, diffuse keratinocyte apoptosis in the basal layer, and subepidermal separation were observed. Due to having apoptotic kerotinocytes and interface tissue pattern which leads to subepidermal separation, our case is regarded as a phototoxic reaction leading to TEN with almost full-thickness epidermal necrosis (Fig. 3). He was managed with intravenous fluids, prednisolone 1.5 mg/kg/day, prophylactic antibiotic, and wet compresses. During follow-up, IVIg was initiated at a dose of 3 g/kg. In a few weeks, with the improvement of cutaneous eruption and hemodynamic stabilization, prednisolone dose was tapered gradually and the patient was discharged. Cutaneous reactions are one of the most prevalent adverse events reported with vandetanib therapy.^1^ Sun exposure has been described as a common triggering factor for cutaneous reactions with vandetanib. In literature, there are few cases of vandetanib-induced photosensitivity reactions including phototoxicity, photoallergic reactions, pigmentation, and SJS.^2^, ^3^, ^4^, ^5^ In the present case, a cutaneous photosensitive reaction was observed. Although our patient did not undergo phototesting, at the beginning symmetrical distribution of the lesions, location only in the sun-exposed areas, appearing immediately after sun exposure led us to diagnose this as a phototoxic reaction. However, in a couple of days lesions progressed to sun-protected areas, with the development of widespread bullous lesions. Our case represents a beginning as a phototoxic reaction induced by vandetanib and then progress to a life-threatening condition, TEN, and only controlled with IVIg. In conclusion, vandetanib can cause various cutaneous side effects from mild, self-limited eruption to severe reactions, which most of them are related to photosensitivity. For that reason, patients should be informed about photoprotection.

**Figure 1:**
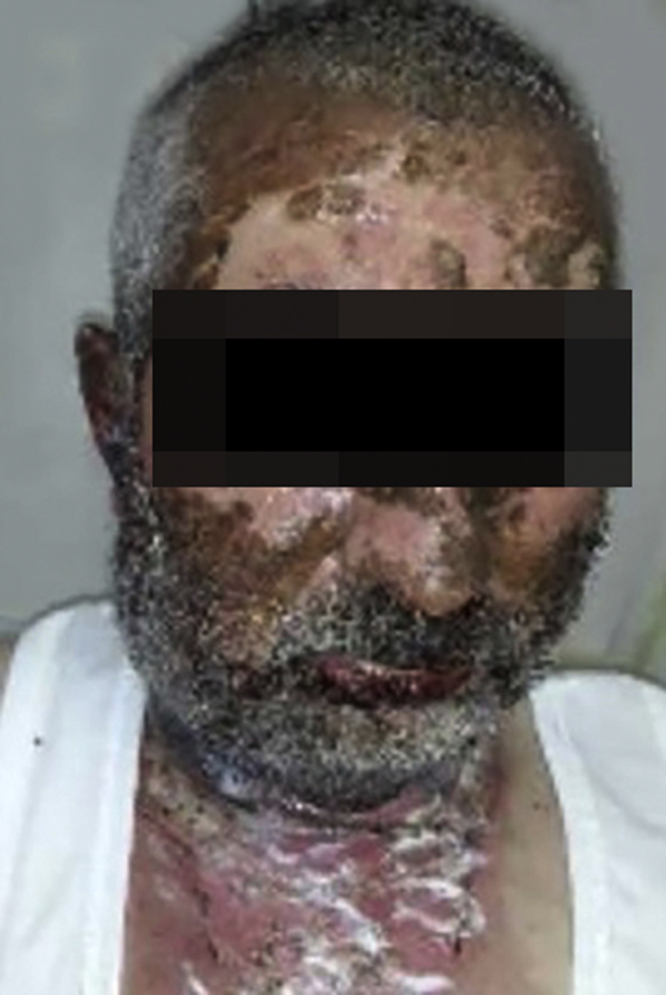
Diffuse epidermal detachment was seen on face.

**Figure 2:**
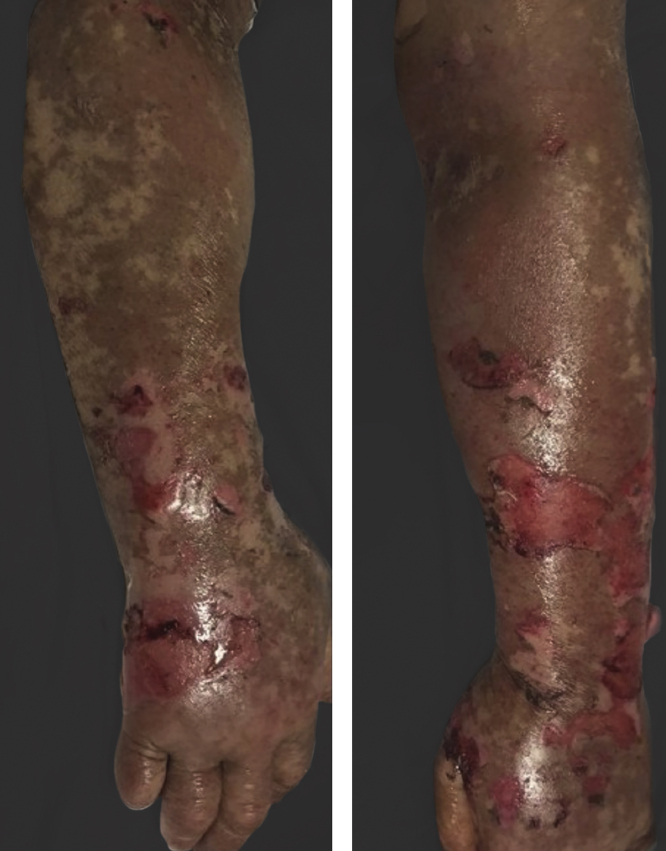
Diffuse epidermal detachment was seen on arms.

**Figure 3:**
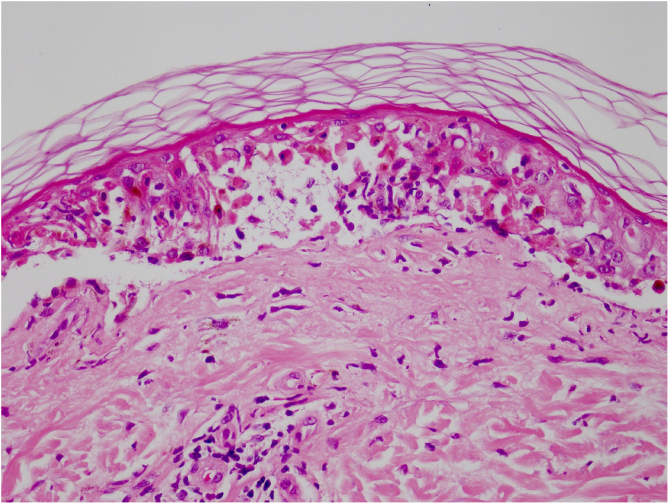
Basal cell vacuolation and subepidermal clivage was seen in light microscopy (Hematoxylin & eosin, ×100).

## Financial support

None declared.

## Authors’ contributions

Busra Demirbag Gul: Collected the data; contributed the data or analysis tools; wrote the paper.

Nilgun Senturk: Collected the data; contributed the data or analysis tools; wrote the paper.

Deniz Baycelebi: Collected the data; wrote the paper.

Levent Yildiz: Collected the data; wrote the paper.

## Conflicts of interest

None declared.
